# Genetic and Epigenetic Characteristics in Isolated Pancreatic Metastases of Clear-Cell Renal Cell Carcinoma

**DOI:** 10.3390/ijms242216292

**Published:** 2023-11-14

**Authors:** Franz Sellner, Eva Compérat, Martin Klimpfinger

**Affiliations:** 1 Department of General, Visceral and Vascular Surgery, Clinic Favoriten Vienna, Kaiser Franz Josef Hospital, 1100 Vienna, Austria; 2 Clinical Institute of Pathology, Medical University Vienna, 1090 Vienna, Austria

**Keywords:** renal cell carcinoma, isolated pancreatic metastases, genetics, epigenetics, seed and soil mechanism

## Abstract

Isolated pancreatic metastases of renal cell carcinoma (IsPMRCC) are a rare manifestation of metastatic, clear-cell renal cell carcinoma (RCC) in which distant metastases occur exclusively in the pancreas. In addition to the main symptom of the isolated occurrence of pancreatic metastases, the entity surprises with additional clinical peculiarities: (a) the unusually long interval of about 9 years between the primary RCC and the onset of pancreatic metastases; (b) multiple pancreatic metastases occurring in 36% of cases; (c) favourable treatment outcomes with a 75% 5-year survival rate; and (d) volume and growth-rate dependent risk factors generally accepted to be relevant for overall survival in metastatic surgery are insignificant in isPMRCC. The genetic and epigenetic causes of exclusive pancreatic involvement have not yet been investigated and are currently unknown. Conversely, according to the few available data in the literature, the following genetic and epigenetic peculiarities can already be identified as the cause of the protracted course: 1. high genetic stability of the tumour cell clones in both the primary tumour and the pancreatic metastases; 2. a low frequency of copy number variants associated with aggressiveness, such as 9p, 14q and 4q loss; 3. in the chromatin-modifying genes, a decreased rate of *PAB1* (3%) and an increased rate of *PBRM1* (77%) defects are seen, a profile associated with a favourable course; 4. an increased incidence of *KDM5C* mutations, which, in common with increased *PBRM1* alterations, is also associated with a favourable outcome; and 5. angiogenetic biomarkers are increased in tumour tissue, while inflammatory biomarkers are decreased, which explains the good response to TKI therapy and lack of sensitivity to IT.

## 1. Introduction

The occurrence of isolated pancreatic metastases of clear-cell renal cell carcinoma (isPMRCC) is rare in the clinical course of clear-cell renal cell cancer (ccRCC). In this entity, the pancreas itself becomes—either definitively or for many years—the sole and only organ site of synchronous or metachronous distant metastases of a ccRCC. If the isolated occurrence of pancreatic metastases (PM) in ccRCC is to be regarded as extremely unusual, the clinical course reveals further peculiarities: (a) In metachronous PM, an unusually long interval from RCC surgery to the occurrence of the PM: from 855 case reports, a mean duration of 9.6 years could be calculated [1], while large institutional reports (N > 20) indicate a time span of 6.9 to 11.2 years (median 9.0 years) [2,3,4,5,6,7,8,9,10,11,12,13,14,15,16,17], with the longest reported interval being 36 years [18]; (b) The high frequency of multiple occurrences of PM: Of 733 casuistic observations, 36.4% concerned multiple PM [1]. This is confirmed in single and multicentre reports with values of 19% to 70% (median 37%) [2,4,6,8,10,12,13,14,16,17,19,20], with a reported maximum of 15 foci [21]; (c) The unusually protracted and favourable clinical course for metastatic ccRCC: For the spontaneous course [22], a 3-year survival rate of 56% was calculated for the few reported, untreated patients (N = 19 [16,23,24,25,26,27,28,29,30,31,32,33,34]). In operated patients, a 5-year survival rate of 75.7% could be determined from 421 case reports [22], and in the single and multicentre reports, the corresponding values are 50–88% [3,4,6,7,8,9,10,11,12,13,14,15,19,20,35,36,37,38,39,40] with a median of 72%. Finally, in patients treated with antiangiogenetic vascular endothelial growth factor receptor tyrosine kinase inhibitors (TKI) [41], a result not significantly different from the operative results was determined [42]; (d) Volume and growth-rate-dependent risk factors generally accepted to be relevant for overall survival (OS) in metastatic surgery are insignificant in isPMRCC [22]: In four large (N > 150) compilations of case reports, singular vs. multiple occurrences, size and number of PMs, as well as synchronous vs. metachronous occurrence and interval to PM occurrence, were not prognostically relevant [22,32,43,44]. An identical lack of prognostic relevance of these risk factors was reported in five large (N > 20) institutional reports [4,8,10,13,14] that analysed exclusively isPMRCC observations.

This overall favourable outcome cannot be explained by the single organ involvement per se, but is a specific feature of the isPMRCC, as evidenced by the significantly worse outcome of single organ metastases of the ccRCC in other organs. In a recent study on the impact of single organ metastases on the course of ccRCC, the median survival time of isPMRCC was three times longer than that of single organ metastases in other organs (8.8 vs. 2.8 years; *p* < 0.001) [45].

Numerous studies have so far dealt with this disease, partly in the form of case studies, or in the form of institutional experience reports, of which 1470 observations were reported by 2022 [1], to which 259 isPMRCC have since been added [2,3,45,46,47,48,49,50,51,52,53,54,55,56], bringing the total number to 1729.

The aim of this review is, therefore, to compile the genetic and epigenetic mechanisms that have become known so far, to be effective in the occurrence of this special metastatic RCC (mRCC) entity.

## 2. Genetic Characteristics and Peculiarities of the isPMRCC

### 2.1. Clear-Cell RCC Genome

The genome of the ccRCC was deciphered as early as 2013 [57]. It is characterized by the biallelic absence or functional inactivation of the *VHL* tumour suppressor gene localized at 3p25 and the frequent inactivation of chromatin-modifying genes, such as *PBRM1*, *BAP1* and *SETD2* [58] (Table 1).

The protein encoded by the *VHL* gene (pVHL) mediates its tumour-suppressive effect by binding to and mediating the proteasomal degradation of the hypoxia-inducible factor HIFα [59,60]. Under physiological conditions, HIFα subunits are unstable and are regulated by cellular oxygen content [61]. The loss or inactivation of *VHL* with consecutive inactivation of pVHL, therefore, leads to the activation and enrichment of HIF despite normoxic conditions and irrespective of the cellular oxygen availability and triggers the subsequent up-regulation of numerous HIF target genes. The activation of these HIF target genes is crucial for the formation and progression of ccRCC due to their role in promoting angiogenesis, tumour cell survival, proliferation and progression. HIFα consists of the subunits 1α and 2α, both of which are involved in ccRCC initiation [60,62]. During further ccRCC progression, however, HIF1α expression (located at chromosome 14q23 [63,64]) is lost in 30–40% since it can act as a tumour suppressor during the progression of ccRCC [60,64]. However, HIF2α acts as an oncoprotein in ccRCC. Due to the behaviour of HIF, two forms of ccRCC can be distinguished: Those in which HIF1α and 2α are overexpressed, and those in which only HIF2α is overexpressed and which are associated with enhanced cell proliferation and unfavourable prognosis [60]. HIF2α-triggered target factors include VEGF-α [60,65], TGF α/EGFR [66], c-Myc [60,67,68], cyclin D1 [69,70], SLC7A5-mTorC1 [60,71,72], GLUT1 [73,74], antioxidant enzymes [75], mitochondrial biogenesis factors [76], GAS6/tyrosine kinase AXL [77] and CXCR4/SDF1 [78], which control critical biological activities such as tumour angiogenesis, cell-autonomous proliferation, increasing glycolysis, resistance to oxidative damage, endoplasmic reticulum stress and metastatic ability [60,67,68,69,70,71,72,73,74,75,76,77,78,79,80,81].

Further frequently altered genes in ccRCC are chromatin-modifying genes: polybromo-1 *(PBRM1*), BRCA1 associated protein 1 (*PAB1*), SET domain containing 2 histone-lysine N-methytransferase (*SETD2*), located on the same 3p chromosomal region [82,83,84], and less frequently, lysine demethylase 5C (*KDM5C*) located on the X chromosome [85] and telomerase reverse transcriptase (*TERT*) promoter located on chromosome 5p [86,87]. The frequency of detectable *VHL* defects is estimated to be up to 90% [58,86,88,89,90]. In contrast, the incidence of the other altered driver genes is significantly lower: *PBRM1* 52.6–26.4%, *SETD2* 35–7.6%, *BAP1* 31–7.5%, *KDM5C* 16–3.8%, *TERT* 14–12.2% and mTor 13–5.7% [58,84,86,88,89,91,92,93,94]. It was soon recognised that these gene alterations are associated with a different tumour biology, and thus, have an influence on the course of the disease and the outcome [90,95]. *PBRM1* is the most frequently mutated gene after *VHL* [84,92] and mutations acquired in this gene largely do not overlap with loss of function mutations in *BAP1* [58,88,90,92,96]. PBRM1 mutations are associated with improved outcome in ccRCC [95,97] and do not correlate with decreased survival [88], whereas the absence of mutations of PBRM1 resulted in worse outcome [90]. *KDM5C* mutations have also been associated with improved clinical outcome in clinical reports [88,94]. In particular, the concurrent mutations of PBRM1 and KDM5C define a subgroup with increased angiogenesis associated with favourable prognosis, as Santos reports [95]. The similar effects of *PBRM1* and *KDM5C* mutations on outcome are consistent with the observation that the vast majority of up- and downregulated genes after suppression of PBRM1 or KDM5C were shared [98]. Conversely, *PAB1* mutations in ccRCC have proved to be a driver of aggressiveness and correlated with reduced outcome [58,84,88,89,90,92,99,100,101,102]. PAB1 mutations further tended to be associated with mTOR mutations [92]. *TERT* and *TP53* were also identified as gene mutations associated with a poor prognosis [58,86,90,99]. However, these gene changes are generally relevant to the occurrence and course of RCC, but none of these changes can be considered specific to the occurrence of metastases, let alone isPMRCC.

**Table 1:** Altered driver genes in ccRCC, metastatic RCC and isPMRCC.

	Altered Genes	References
Clear cell RCC	VHL Gen	[57,58,82,88]
	Chromatin modifying genes: e.g., PBRM1, BAP1, SET2, KDM5C	[83,86,88,89,92,94]
	Further driver genes: e.g., pTEN, TERT, p53	[86,89,92,93,94,99]
Metastatic RCC	Loss of 9p, 14q Number of somatic copy number variants in primary ↑ metastatic potential ↑: low ITH ^1^ and high SCNA ^2^ in primary	[63,103,104]
isPMRCC	9p loss missing Number of somatic copy number variants ↓ chromatin-modifying genes: PBRM1 ↑, BAP1 ↓, KDM5C ↑ High genetic stability, constrained evolutionary process	[50,91,103]

**↑** increased, **↓** decreased, ^1^ intratumoural heterogeneity, ^2^ somatic copy number alterations.

### 2.2. Genetic Profile of Metastatic ccRCC

For the question of possible genetic characteristics of the isPMRCC, studies that specifically investigated genetic alterations that control and influence the metastatic behaviour of the RCC are therefore more relevant. Such a study was conducted and presented for the first time in 2018 by Turajlic [103]. In this groundbreaking analysis of 575 primary and 335 metastatic biopsies across 100 patients with metastatic ccRCC, the authors were able to identify three genetic changes that shape the metastatic behaviour of the RCC: 1. the loss of 9p21.3 and less pronounced 14q31.2 are hallmark genomic alterations at the beginning of the metastasis process; 2. the metastasis potential of RCC is reduced by low intratumoural heterogeneity and a small proportion of somatic copy-number alterations; and 3. distinct patterns of metastasis are caused by punctuated and branched evolution (Table 1).

### 2.3. Genetic Profile of isPMRCC

So far only three publications have been presented in which this particular problem is addressed [50,91,103]. On the one hand, this is an inevitable consequence of the extreme rarity of isPMRCC, but on the other hand, it is also due to the fact that techniques such as next-generation sequencing have only been developed and used in recent years [82] (Table 1).

**Figure 1:**
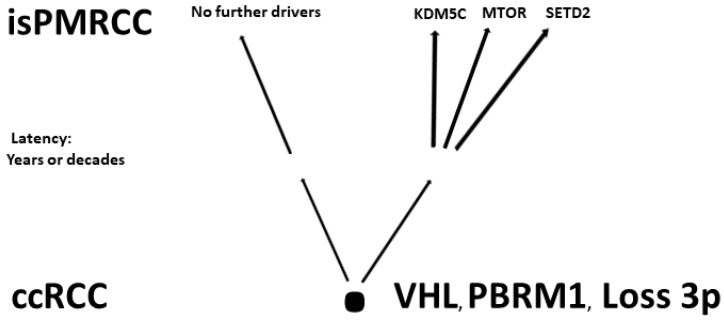
Involved genes in isPMRCC.

(a) In the already cited work of Turajlic [103], among the 100 patients, there were also three isPMRCC observations, whose genetic profile was analysed and presented in detail for the first time. The isPMRCC showed an independent genetic profile characterized by the absence of 9p loss and a significantly lower genome instability index: Despite a 15-year and 8-year interval between primary ccRCC and clinical manifestation of PM, only one additional driver mutation was observed in two cases (mTor and *SETD2*, respectively) and in the third case, even after 17 years, there was no additional driver event to prove.
(b) Based on the improved prognosis of multiorgan metastases of ccRCC with concurrent PM compared to cases without PM, as shown by Grassi [105], and since repeatedly confirmed [11,91,97,106,107,108,109,110], Singla and colleagues in 2020 focused on the question of genetic characteristics of PM in mRCC [91]. (Their study group included 31 patients, but only a subgroup of just 10 (32%) met the isPMRCC criteria. However, the larger group (68%) experienced PM with simultaneous extrapancreatic multiorgan metastases of the ccRCC, which needs to be considered when assessing the relevance of the results for the specific isPMRCC topic discussed here because the detailed differences in metastasis behaviour between the two groups (single organ vs. multi-organ metastases) and the very special clinic of the isPMRCC (9.5 years metastasis-free interval until occurrence of PM and 75% 5-year survival rate, Section 1) make some genetic/epigenetic differences at least possible). In their extensive, meritorious study, Singla and colleagues were able to document genetic changes associated with less aggressive disease pathways: a low frequency of copy number variants associated with aggressiveness, such as 9p, 14q and 4q loss [63,103,104]. Furthermore, the authors found a low rate of *PAB1* (3%) and a high rate of *PBRM1* defects (77%)—changes associated with a less aggressive disease course [96,111]. Similarly, no driver mutation could be detected in *TERT*, which is associated with an aggressive disease course in RCC [86]. In contrast, *KDM5C*—after *VHL* and *PBRM1*—was the third most common gene mutation in the studied material with a frequency of 24%. As already pointed out above (Section 2.1), the concurrent occurrence of *PBRM1* and *KDM5C* mutations is again a sign of a favourable course [95]. The high frequency of *KDM5C* mutations differs from metastatic ccRCC without PM in two respects. On the one hand, the value of 24% is the highest reported frequency so far [84,88,89,92]. On the other hand, in non-isPMRCC studies, *KDM5C* was only the fifth most common mutation [58,84,88,89,92,94]. As a further important characteristic of PMRCC, these authors also stress the unusual genetic stability of tumour cells, as limited diversification was observed both in the primary tumours leading to PM and in the subsequent PM themselves. The authors concluded that tumours and metastases from patients with PM are consistent with a constrained evolutionary process.
(c) Finally, Lou presented in 2023 an isPMRCC [50] that showed in the next-generation sequencing, three gene mutations (*VHL*, *PTEN*, *KDM5C*), a low tumour mutation burden and a microsatellite stable status. The fact that of the chromatin-modifying factors, only KDM5C was mutated is striking, as it further confirms Singla’s result of an increased frequency of KDM5C mutations (Figure 1).

The observations made so far at the isPMRCC can be summarised by three characteristics (Table 1): 1. a lower number or absence of copy number variants associated with increased aggressiveness (e.g., 9p, 14q, 4q); 2. an evolutionary profile characterized by the rare occurrence of mutations associated with aggressive pathways, such as BAP1 and TERT, and the increased occurrence of beneficial gene mutations, such as PBRM1, leading to a specific genetic profile that is further accentuated by the increase in KDM5C mutations observed in two of the three studies; and 3. high genetic stability.

Further evidence for the high genetic stability of this entity is provided by reports of subsequent tumour progression after isPMRCC therapy, which occurred in 43% of 288 case reports after a median interval of 29.8 months [106], and 15.3% of these appeared as further isPMRCC in the pancreas remnant [2,20,30,46,56,112,113,114,115,116,117,118,119,120]. In institutional communications, the incidence of newly isolated PM is estimated to be between 9% and 62% (median 27%) [2,3,4,7,9,10,12,45,112,113].

## 3. Epigenetics of isPMRCC

### 3.1. The Impact of a “Seed and Soil Mechanism” in isPMRCC

While the above-cited genetic studies have been able to identify several factors that are at least co-responsible for the unusually favourable outcome, the question of the causes of the sole and exclusive single-organ involvement of the pancreas (organotropism) remains unanswered at present. Due to the exclusive rarity of the isPMRCC, no working group has yet specifically investigated this issue.

The theory that innate or acquired direct lymphatic or venous vascular connections between the kidney and pancreas are responsible for the occurrence of PM [23,24,121,122,123,124,125,126,127,128]—initially derived from a few individual cases—was refuted by the results of later more extensive studies. A high importance of this mechanism would imply that left-sided RCCs should preferably lead to metastases in the near pancreatic tail and corpus, whereas right-sided RCCs should preferably metastasize in the near pancreatic head. In other words, this local metastasis mechanism should inevitably result in a dependence of the localization of metastases in the pancreas from the side of the primary RCC. However, as our working group demonstrated for the first time in 2006 and was shown subsequently in increasingly large literature compilations [1,106,120], the PMs are independent of the ccRCC side, being evenly distributed over the pancreas. This even distribution has now been confirmed and documented in numerous institutional studies [3,10,13,14,31,129,130,131,132,133]. However, to the best of our knowledge, the well-documented even distribution can only be explained by a systemic hematogenic metastasis pathway. The fact, in turn, that after systemic hematogenic tumour cell dissemination in all organs, manifest metastases occur only in the pancreas is then only conceivable if the embolized tumour cells have a special affinity for the pancreas. This means that the phenomenon of the isolated occurrence of PM is obviously based on an exclusive “seed and soil” mechanism (SSM), which allows the growth of embolized tumour cells only and exclusively in the pancreas while in all other organs, the formation of metastases is impeded [134,135].

The SSM [136,137,138] was identified and described by Paget as early as 1889 [139]. This SSM explains that the distribution pattern of metastases is not a uniform, random pattern. On the contrary, the individual primary tumour entities are assigned preferred host organs because the definitive metastatic settlement is the result of absolutely necessary multistage cascade-like interactions of cancer cell properties (seed) with those of the host organ (soil). Each host organ places different demands on embolized cancer cells. The growth of embolized tumour cells to clinically manifested metastases is therefore only possible in organs in which the corresponding characteristics of the host and tumour cell exactly match each other because the blockage of a single stage of this complex process can make metastasis formation impossible [140,141,142]. In the majority of cases, SSM leads to a relative advantage or disadvantage of metastasis formation in potential host organs. In the case of isPMRCCs, however, a highly specific SSM is present, which allows metastasis formation solely in the pancreas but absolutely prevents it in all other organs. However, this “absolute” effective SSM is able to explain the lack of meaningfulness of metastasis volume and growth-rate-dependent risk factors for OS mentioned at the beginning of this section. All these risk factors are only an expression of the magnitude of the risk that further occult micrometastases are already present outside the pancreas at the time of PM surgery, leading later to tumour progression. However, since the exquisite SSM does not allow embolized tumour cells to survive outside the pancreas—or, as is equally conceivable, that forces the embolized tumour cells definitively, or at least for many years, into a dormant, non-growth state [143,144,145]—this risk is or tends to be zero and the risk factors must remain ineffective.

### 3.2. Epigenetics and SSM in isPMRCC

Epigenetic markers, based on DNA-methylation, histon modifiers and micro RNA expression jointly control gene expression in RCC [146]. In RCC, for example, aberrant promoter methylation in more than 200 genes and more than 120 deregulated miRNA were reported as early as 2017 [146,147]. DNA methylation plays a significant role in the regulation of gene expression, e.g., gene promoter methylation, that silences its corresponding gene expression. In 2019, Nam reported that the gene signature related to DNA methylation differs between primary RCC and RCC metastases, as it was found, that metastatic tumours often demonstrated more pronounced changes compared to primary tumours, e.g., in metabolism-related HK2 and SZC16A3 [148]. Several HIF-target genes were hypomethylated with increased expression in metastatic RCC, including ADM, TNFAIP6, CAV1, HK2 and ALDOC. Conversely, promoter hypermethylation with silencing of the corresponding genes was identified, e.g., in the gene encoding estrogen-related receptor γ—an activator of transcription—with the strongest reduction noted in metastatic RCC. These results provide evidence that in relation to DNA methylation, metastatic RCC has a specific pattern compared to the primary RCC. Micro-RNA in turn, to give another example, control cancer metastasis, because of their ability to inhibit target genes involved in different steps of cancer metastasis cascade, e.g., EMT, migration and metastasis settlement [149,150,151,152,153,154,155,156]. In addition, it was demonstrated that the miRNA profile differs between metastatic and non-metastatic RCC [157,158,159], just as it is influenced by the location of metastases in RCC [160].

For the metastatic RCC (mRCC), a specific pattern regarding DNA methylation or miRNA profile is documented in the literature. However, to the best of our knowledge, no epigenetic study has so far been presented for the extremely rare isPMRCC. This makes it currently impossible to compare epigenetic changes in isPMRCC with other RCC entities. Therefore, it remains completely open whether and which specific epigenetic changes are characteristic of the occurrence of isPMRCC.

The exact cause(s) of the highly specific SSM in the isPMRCC has not yet been investigated and explained due to the rarity of this entity. Therefore, at present, only those epigenetic mechanisms that have been identified as triggering organotropism in more frequent and, therefore, better-investigated tumour entities can be put up for discussion (Table 2): (a) the premetastatic niche; (b) the chemokine receptor/ligand mechanism; (c) the effects of metabolic adaptation; (d) the immuno-surveillance; and (e) the impact of micro-RNA (miRNA)

(a) The premetastatic niche (pMN) is the result of the ability of tumours to manipulate a host organ prior to the formation of metastases in such a way that a special microenvironment is created that allows the subsequent successful metastasis settlement, by inflammation, immunosuppression, enhanced angiogenesis, vascular leakiness and extracellular remodelling [142,161,162,163]. Since the formation of pMN results from the interaction of primary tumour-derived components (tumour-derived secreted factors including VEGF, TNF-α, TGF-β, G-CSF and tumour-derived extracellular vesicles (EV) like, exosomes, microvesicles containing a variety of proteins, mRNAs, miRNAs and signalling molecules) with tumour-mobilised bone-marrow-derived cells (MDSC, TAM) and the local microenvironment [163,164,165,166,167,168,169,170,171,172,173,174,175], this is associated with organotropism, which is a characteristic of pMN [163]. In the ccRCC, the formation of a pMN in the lung was described in 2011 [175]. However, pMN formation of RCC in the pancreas has not been reported;
(b) Successful chemokine receptor/ligand reaction is a necessary requirement for the activation of numerous signal-transforming pathways. These are critical in the early metastatic process [176,177]. Signalling between chemokines and their receptors regulates tumour cell settlement in host organs e.g., by recruitment of MDSCs, TAMs, Tregs and tumour-associated neutrophils into distant secondary sites, and thus, supporting the formation of the premetastatic niche [162,163], or in supporting cancer by stepwise activating the pluripotency regulator transcription factors OCT4, NANOG and SOX2, whose activation helps cancer cells in attaining stemness properties [176,178]. Thus, they are considered critical regulators of self-renewal and pluripotency that mediate tumour proliferation, differentiation, metastasis and prognosis [176,179,180]. With RCC, CXCL6/7- and CXCL12-mediated activation of CXCR1/2 and CXCR4 is documented [176,179,181]. Since the chemokine receptor is specific to the tumour cell and the ligand is specific to a host organ, a successful interaction will only take place in those tissues where the receptor and ligand exactly match each other. This inevitably leads to organotropism in metastasis formation. The effect of this mechanism on metastatic behaviour was demonstrated early in breast cancer: e.g., breast cancer cells express high levels of CXCR4 and CCR7, which are responsible for metastasis settlement in LN, lung, liver and bone marrow, as these organs are rich in corresponding ligands CXCL12 and CCL21 [182,183];
(c) The impact of metabolic adaptation
  At the stage of early, avascular growth, micrometastases pass through a critical phase, as the supply with energy carriers is limited by diffusion alone [136], e.g., 85–100 μm away from tumour vessels, hypoxic cells are already detectable [184]. Therefore, those cell clones will preferably be able to survive this stage and are able to optimally utilize all the locally available energy carriers by bypassing metabolic barriers by metabolic adaptation, so that the tumour cells acquire a metabolic signature adopted for survival at a particular metastatic site [185,186]. Here again, a successful interaction between a host organ that provides the energy carriers and the tumour cells that can utilize the energy carriers is a necessary prerequisite for metastasis formation–i.e., an SSM mechanism, which again, triggers organotropism in metastasis.
  In the case of the isPMRCC in particular, however, an additional metabolic mechanism has to be considered. Rapid tumour growth is usually accompanied by increased metabolism, which affects the microenvironment. Critical blood flow with hypoxia, but especially the Warburg effect (aerobic glycolysis as part of tumour-specific metabolic reprogramming despite the presence of oxygen and functionating mitochondria) leads to increased glycolysis in the tumour with the accumulation of acidic lactic acid [187,188]. As a result, the tumour cells modify the microenvironment to an acidotic pH [187,189,190]. This, in turn, gives an advantage to tumour cell clones that are adopted to acidic pH values as low pH reinforces the metastatic potential of tumour cells by relaxing cell–cell contact, by degrading extracellular matrix, by fostering tumour cell migration and by suppression of anti-tumour immunity [188,191,192,193]. In the case of slow-growing isPMRCC, however, acidosis caused by rapid tumour growth cannot be of particular importance. On the contrary, the isolated growth of cells in the pancreas at least suggests the presence of cell clones that are well adapted to an alkaline environment. This would inevitably lead to an organotropism in the pancreas that is characterised by an alkaline environment, whereas in extrapancreatic organs, the formation of metastases is impeded [1];
(d) Immuno-surveillance
  The importance of the immune system in mRCC was early assumed by the rarely observed phenomenon of metastases spontaneous regression [194,195,196,197], also in the pancreas [25], as the cause of which spontaneous changes in the immune system were correctly assumed. The ability to evade the immune system through specific inhibitory signalling pathways such as T-lymphocyte-associated protein 4 (CTLA-4) and programmed cell death protein-1 pathways (PD-1/PD-L1) is a fatal hallmark of tumours [100,198,199]. This knowledge led to the development of immunotherapy (IT) with the use of monoclonal AK (anti-PD-1 nivolumab and anti-PD-L1 avelumab) and monoclonal AK against CTLA-4 (ipilimumab). Since blockade of the immune system also plays an important role in the progression of mRCC, IT is generally effective in advanced ccRCC [200,201]. It is, therefore, all the more remarkable that in Singla’s study, IT was found to be ineffective in PM of the ccRCC, whereas TKI therapy was effective [91]. This unexpected result could be explained and supported by the behaviour of biomarkers. While angiogenetic markers were elevated (e.g., enrichment of endothelial cells, low frequency of macrophages, B cells, T cells, natural killer cells and neutrophils and marked BPRM1 loss), inflammatory markers remained low, making ccRCC with PM appear to belong to the angiogenetic non-inflammatory subtype of mRCC [202,203]. This leads to the conclusion that in ccRCC with PM, the tumour cells are recognized as “foreign” and fought against, so an additional IT does not bring benefit. Of course, it remains unknown why of all things and why only in one single organ, the pancreas, the immune defence is ineffective, and thus, triggers an organotropism. Conversely, the high presence of angiogenetic biomarkers in isPMRCC shows the high importance of angiogenetic mechanisms in this entity and explains the high sensitivity to TKI treatment [41,42,91,201,204,205,206,207,208];
(e) Importance of miRNA
  miRNAs are a class of small (16–22 nucleotides [209]) non-coding regulatory RNAs that negatively regulate the expression of target genes by translational repression or degradation of mRNA [151,156,160,210,211]. They are involved in carcinogenesis as they are associated with the activation of proto-oncogenes or inactivation of suppressor genes [156,209]. miRNAs are also able to regulate cancer metastasis due to their ability to inhibit numerous target genes involved in different steps of cancer metastatic cascade [151,156], such as EMT [151,156], migration, settlement and proliferation of embolized tumour cells [156,159,212]. The so-far discovered varieties of miRNAs with altered and disturbed expression in RCC, which regulate carcinogenesis but also the different steps of the cancer metastatic cascade [150,152,153,155,159,209], are certainly accompanied by a large number of heterogenous tumour cells. This favours the formation of metastases as this increases the likelihood of “matching” cancer cells reaching a potential host organ. Furthermore, it was demonstrated that the miRNA profile differs between non-metastatic and metastatic RCC [157,159,160] and that there is also a dependence of the miRNA profile from the host organs affected by metastasis [160]. These results may indicate an interrelation between the miRNA profile and the ability to metastasize in different host organs in mRCC, which could cause organotropism in metastatic settlement. The fact that an SSM triggered by the profile of miRNAs (transported by EV to the potential premetastatic sites, see Section 3.2 (a) can in principle occur is documented in the literature, at least for more common and, therefore, better-researched tumour entities such as breast cancer metastases [213,214].

**Table 2:** Mechanisms leading to organotropism in metastasis settlement.

Mechanism	References
Pre-metastatic niche	[142,161,162,163]
Chemokine receptor mechanism	[176,177,178,179,180,181]
Metabolic adaptation of tumour cells	[185,186,187,188,189,190,191,192]
Differences in Immunosurveillance	[194,195,196,197,198,199,200,201,202,203,204]
Micro-RNA profile	[151,152,153,154,155,156,157,158,159,160,210,214,215]

## 4. Conclusions

In isPMRCC, research in recent years has uncovered numerous genetic and epigenetic mechanisms that can explain the unusually protracted and favourable course and the specific response to drug therapy: e.g., high genetic stability, low frequency of copy number variants, a profile of chromatin modifying genes alterations associated with favourable course [*PBRM1* ↑, *PAB1* ↓) and affiliation to the angiogenetic subtype. Whether our increasing knowledge of the genetic and epigenetic characteristics of the exquisitely rare isPMRCC will help to show a relationship of radiomic features with genetic mutations status as *VHL*, *PBRM1*, *PAB1*, *KDM5C*, *SETD2* or expression of miRNA [215,216,217,218,219,220,221], will have to be shown in future studies; this also applies to the influence of therapy outcomes by external factors [222]. However, such studies are hampered by the rarity of isPMRCC, as meaningful collectives are only possible through large multi-institutional studies or extensive literature compilations. The extremely unusual behaviour of the isPMRCC leads to the conclusion that further hitherto undiscovered biological mechanisms are involved. Therefore, investigations of this unusual entity may be useful in the therapeutic debate, which currently revolves around the optimal use of angiogenesis inhibition and IT in mRCC [91]. However, the cause of the isolated occurrence of PM in isPMRCCs is still unknown. The uniform, long-term constant clinical course suggests at least that the phenomenon of isPMRCC is also based on uniform pathomechanisms. Therefore, genetic studies appear appropriate to clarify the mechanisms that cause the exclusive occurrence of pancreatic metastases and trigger their absence in all other organs. This could both lead to a better understanding of the complex metastatic process and help achieve the goal: to hamper the metastatic process.
